# Rib pseudoarthrosis with thoracic outlet syndrome in pediatric gymnast: a case report

**DOI:** 10.1186/s13256-023-04182-8

**Published:** 2023-11-30

**Authors:** Jessica Kraft, Ann L. Contrucci

**Affiliations:** Philadelphia College of Osteopathic Medicine Georgia, 625 Old Peachtree Rd NW, Suwanee, GA 30024 USA

**Keywords:** Pediatric, Pseudoarthrosis, Thoracic outlet syndrome, First rib

## Abstract

**Background:**

This case study evaluates the diagnosis and treatment of a 12 year old Caucasian male gymnast who had several diagnoses including an isolated first rib fracture, resultant pseudoarthrosis of the first rib, and the development of symptomatic thoracic outlet syndrome. We discuss the causes, prevalence, and suggestions for prompt diagnosis and treatment of these conditions in pediatric patients. Although all three conditions are rare in a child, this case highlights the importance of having a high clinical index of suspicion in recurrent pain in pre-pubertal athletes.

**Case presentation:**

A 12 year old Caucasian male underwent several years of conservative treatment with physical therapy and rest without resolution of his left shoulder pain. He was subsequently diagnosed with pseudoarthrosis of the first rib and thoracic outlet syndrome, which was curative by surgical removal of the first rib, and allowed him to return to his baseline activity level.

**Conclusions:**

Since each of these diagnoses are rare, especially in the pediatric population, we aim to educate the medical community on the prompt diagnosis and treatment of these conditions.

## Background

A 12 year old male gymnast with a two year history of left shoulder pain, since the age of 10, was diagnosed with pseudoarthrosis of the left first rib and resultant thoracic outlet syndrome, including muscle atrophy and brachial plexus weakness. After failure of conservative treatment with physical therapy and rest, he underwent excision of the left first rib. His symptoms resolved and he was able to return to competition level gymnastics six weeks later.

## Case

This is an initial presentation at age 10 of a Caucasian male who presented with a two to three month history of worsening left shoulder pain at the time of his first visit to a physician. Past medical history was positive only for seasonal allergies and he did not have a history of injuries. The patient was a competitive gymnast who practiced approximately ten hours per week. His shoulder pain was accompanied by a "tic" type movement consisting of hyperextension of the left shoulder multiple times per day. The patient said this movement was to "relieve the tightness" in his left shoulder. The patient did not complain of any left arm weakness at this time. Initial treatment included NSAIDS and rest with minimal improvement in symptoms.

**Fig. 1:**
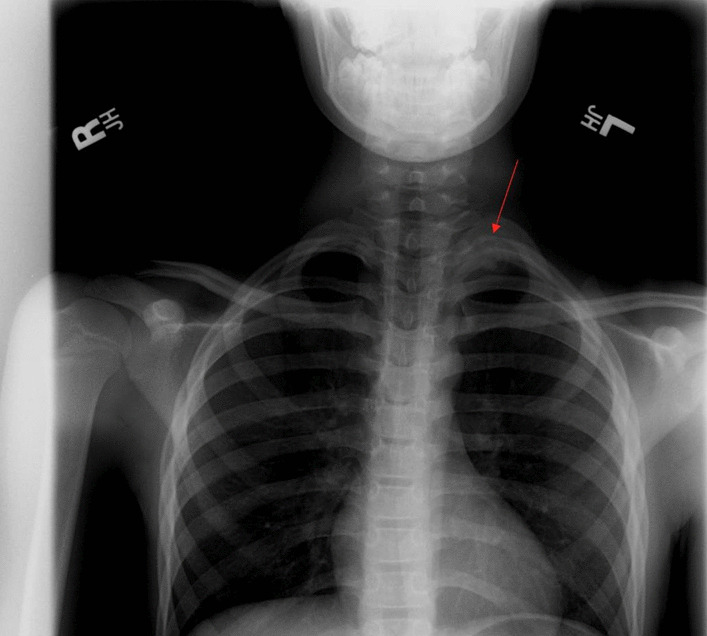
Initial chest X-ray with evidence of left first rib pseudoarthrosis (red arrow is pointing to the pseudoarthrosis present in the patient’s first rib)

**Fig. 2:**
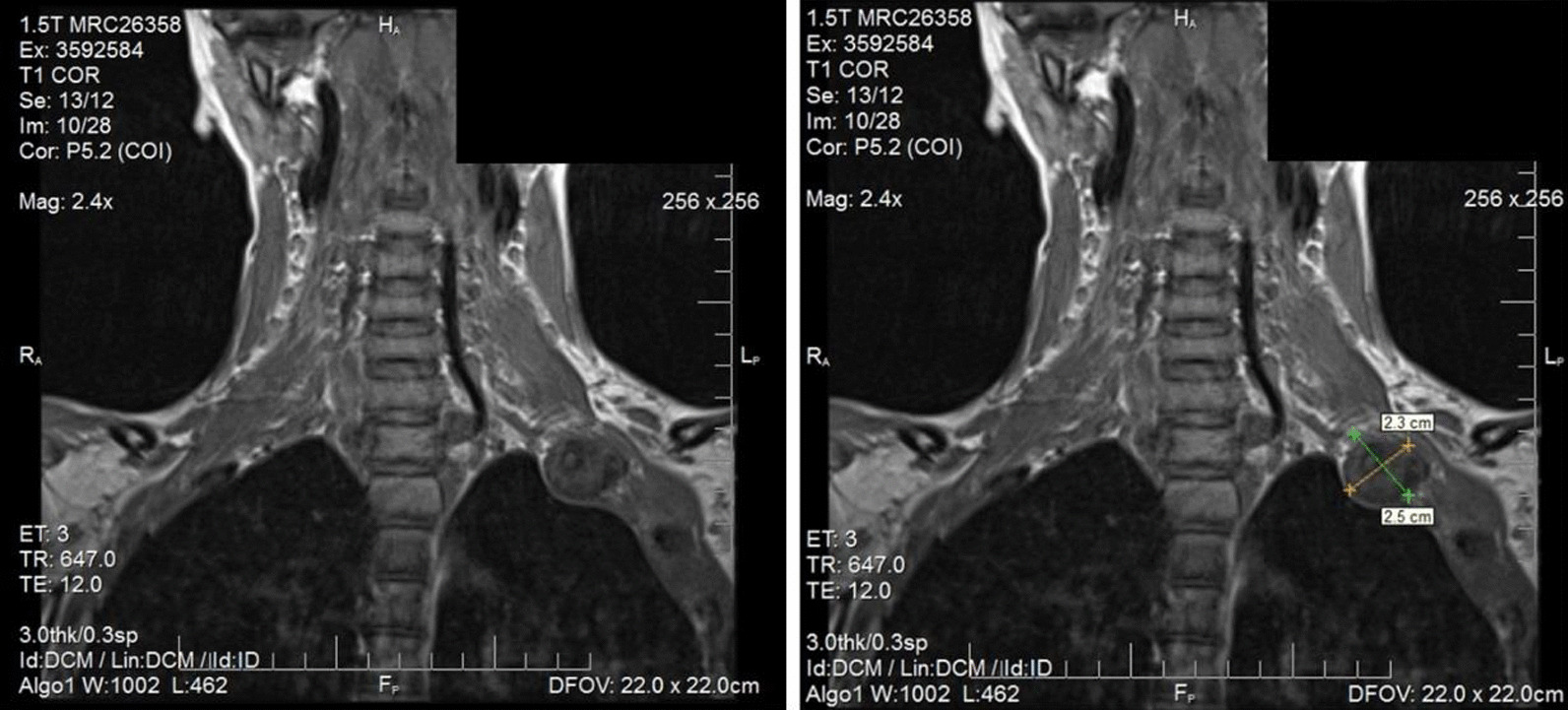
Coronal view of cervical magnetic resonance imaging (MRI) showing pseudoarthrosis of left first rib

The patient was seen by a pediatric orthopedic surgeon who diagnosed him with "overuse syndrome" and prescribed physical therapy. There were no imaging studies conducted at the initial visit except an X-ray per the mother’s request, who was a pediatrician, and was read as a normal film with no significant findings (Fig. 1). A brief focused musculoskeletal exam was performed on the initial visit. After two months of consistent physical therapy, the patient returned to competitive gymnastics. This cycle continued every few months for two years, with the patient's shoulder pain and tightness always returning after a period of physical therapy and rest. Each time, the patient returned to the pediatric orthopedic surgeon and he continued to be diagnosed with persistent "overuse syndrome" with the recommendation to restart physical therapy; no comprehensive physical exam nor imaging studies were done on any subsequent visits. At the age of 12, after multiple cycles of the above, the patient had a final round of intense physical therapy for three months at three times per week, and complete upper extremity rest. Two weeks after the patient returned to gymnastics, he experienced severe left shoulder pain and worsening of the "tic" movements. At this time, the patient's mother noticed atrophy to the left upper scapula region and vague weakness of the left upper extremity. The patient's pediatrician agreed to an MRI of the cervical spine (Fig. 2).

**Fig. 3:**
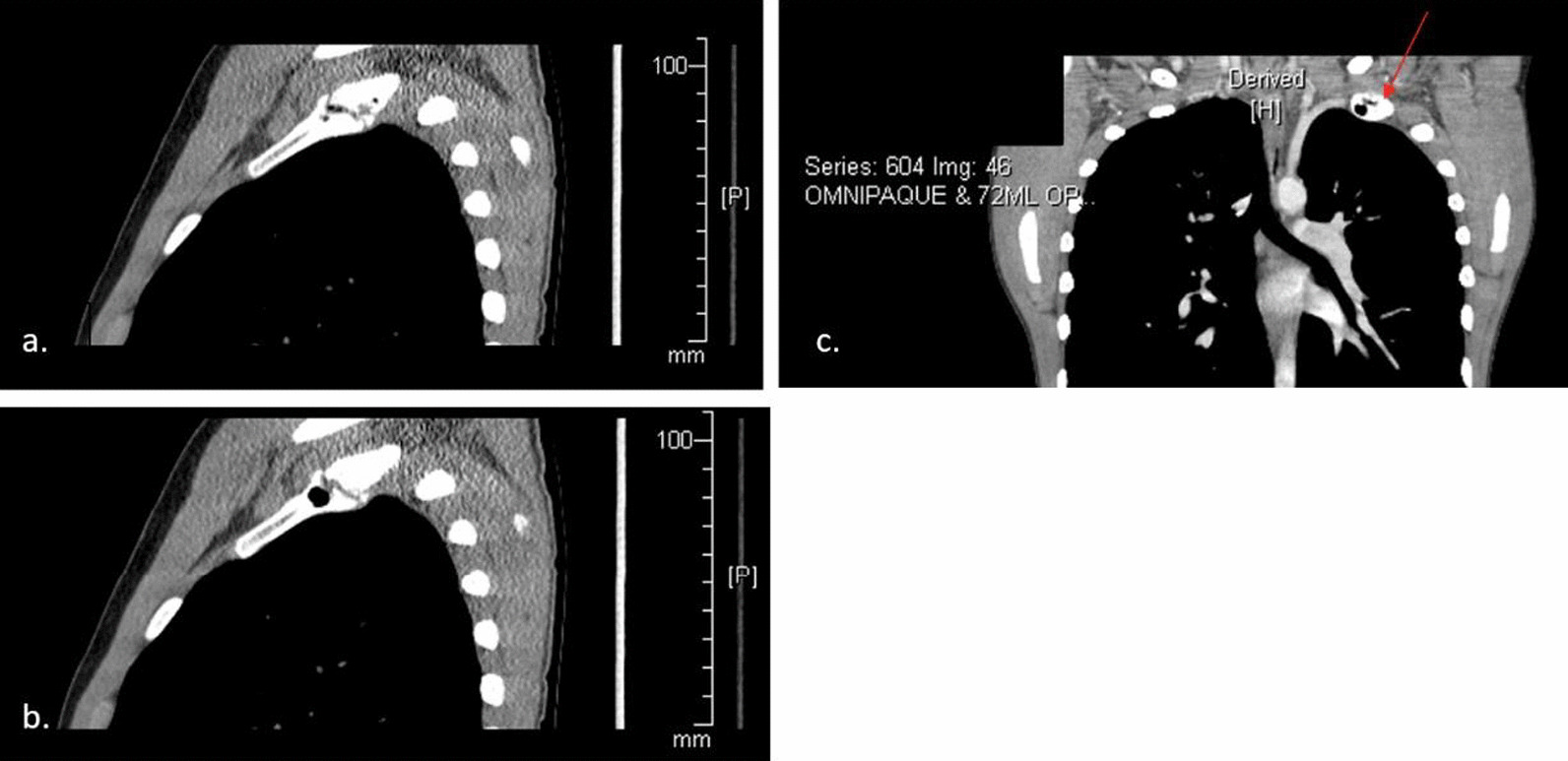
**a**, **b** sagittal computed tomography (CT) showing non union of first rib. **c** coronal CT exhibiting pseudoarthrosis of left first rib with vacuum phenomenon. The red arrow shows the accumulation of gas noted in the left first rib. This is demonstrating the pseudoarthrosis and vacuum phenomenon from the unhealed fracture

**Fig. 4:**
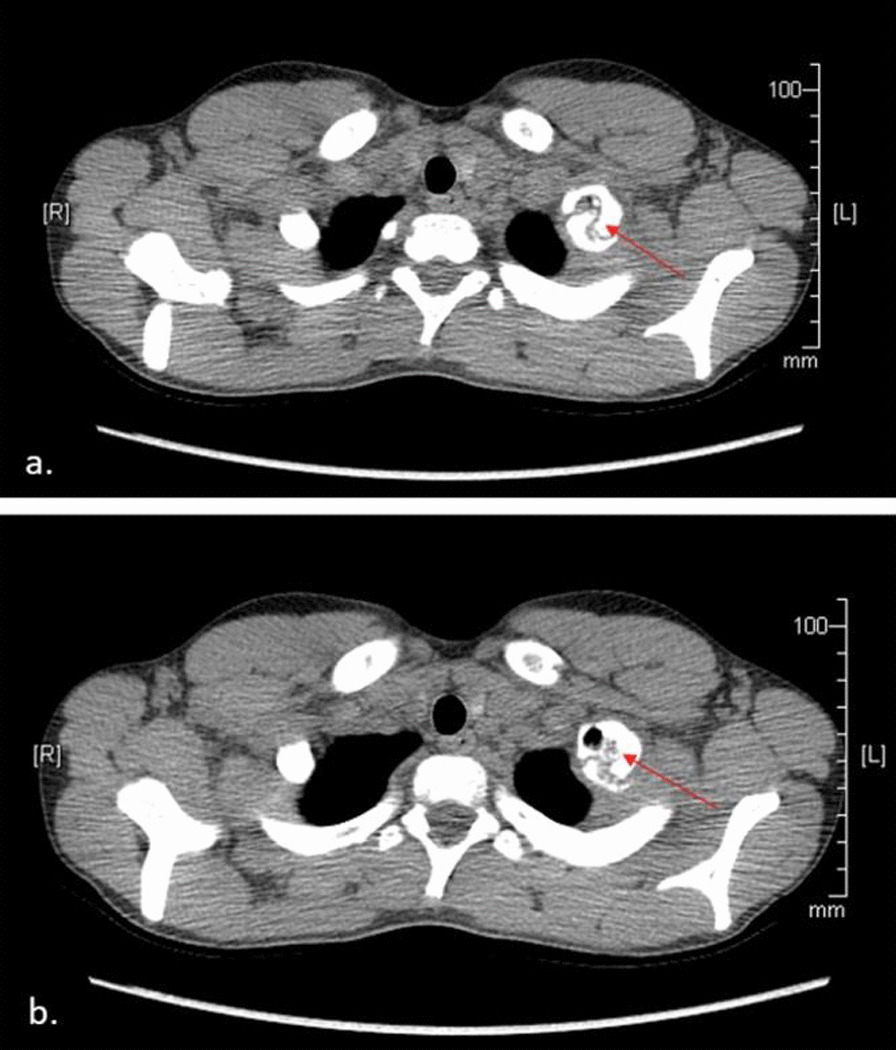
** a**, **b** Axial computed tomography (CT) scan revealing pseudoarthrosis of left first rib with vacuum phenomenon (red arrow is pointing to the pseudoarthrosis present and the vacuum effect on the first rib)

**Fig. 5:**
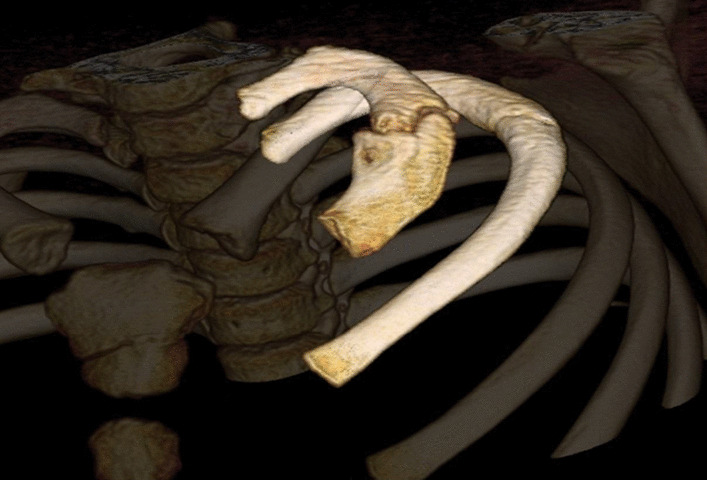
Computed Tomography bone reconstruction showing pseudoarthrosis of left first rib that compressed the brachial plexus resulting in thoracic outlet syndrome. Note left second rib with healing fracture

The MRI is shown in Fig. 2 and was read as: “unusual nodular mass at the apex of the left hemithorax involving the antero-lateral aspect of the left first rib. There is less well-defined contiguous soft tissue thickening associated with the left second and third ribs. Imaging characteristics are nonspecific. Tumor cannot be excluded. No abnormality of the cervical or thoracic spine is seen.” Chest CT was recommended for further evaluation. Chest CT was performed within a day of the MRI and is shown in Figs. 3 and 4. Chest CT was read as: “multiple lucencies demonstrated in the lateral aspect of the left first rib with an irregularly shaped and somewhat sclerotic appearing fracture plane. There is evidence of vacuum phenomenon in the anterior fracture fragment. Findings overall are suspicious for pseudoarthrosis. Mottled lucencies are present in the left second lateral rib. There is associated nonspecific soft tissue thickening extending from the first to the second and third ribs. Given the combination of findings, differential diagnosis includes chronic stress reaction versus infiltrative tumor.” Additional 3D reconstructed CT images were done showing the first rib pseudoarthrosis as well as demonstrating a non-displaced fracture through the left second rib (Fig. 5).

After these radiologic findings were reviewed, the patient was seen by multiple pediatric specialists including pediatric oncology, orthopedic oncology, and pediatric orthopedics. Labs were done and all were normal. Upon the mother consulting with an orthopedic surgeon who had authored a case report 20 years prior involving a female gymnast with a first rib fracture who subsequently underwent first rib resection [4], the patient was referred to a thoracic surgeon who recommended first rib resection.

The patient underwent a left first rib resection without complication. He recovered well post operatively, and the pain, “tic”, and atrophy drastically improved. Follow up several years later revealed a smaller left second rib pseudoarthrosis on CT, with occasional symptoms of pain in the left shoulder area but no further intervention was recommended.

## Discussion

Due to the shape and location of the first rib, fractures are uncommon, especially in the pediatric population [1, 4]. An isolated first rib fracture is extremely rare in a traumatic event and usually is accompanied by fractures of other surrounding structures [4]. Stress fractures of the first rib are most commonly due to the contraction of the scalenus anterior muscle that attaches to the cranial surface of the first rib [4, 6]. As children grow, bone stiffness increases, and their bones are more likely to buckle under impact. In addition, during growth spurts, children are less flexible due to the increase in bone length and are more prone to injuries [2]. Our patient's first rib fracture may have been due to the intense gymnastics training; however the patient did not recall a specific traumatic injury. Children, especially in high impact sports such as gymnastics, who present with recurrent shoulder pain not relieved by physical therapy should have imaging to rule out a stress fracture.

First rib fractures generally heal with rest and without complications. If late complications do occur, they may include Horner’s syndrome, thoracic outlet syndrome, and pseudoarthrosis [6]. Rarely does a patient develop nonunion of a first rib fracture, and if so, they are historically asymptomatic and do not require treatment. However, our patient did develop symptomatic nonunion of first rib fracture requiring treatment. Proffer et. al., reported a female gymnast with a two year duration of stabbing shoulder pain due to nonunion of a first rib fracture. That patient did not have any deformity or muscle atrophy due to the nonunion. After the patient failed conservative treatment, they proceeded with the excision of the first right rib, and the patient’s symptoms resolved post operatively [4]. This is consistent with our patient’s final outcome.

Our patient developed symptomatic thoracic outlet syndrome as a result of the nonunion of the left first rib. Thoracic outlet syndrome is a group of neuropathic symptoms resulting from the compression of the brachial plexus. These symptoms include pain, numbness, tingling, or atrophy of the upper extremities. The brachial plexus, subclavian artery, and subclavian vein lie between the clavicle and the first rib. These structures are easily compressed with abnormalities of the first rib, as seen in our patient with pseudoarthrosis of the first rib. Conservative treatment of thoracic outlet syndrome includes activity modifications, physical therapy, and rehabilitation. If conservative treatments do not resolve symptoms, surgical removal of the first rib is recommended to decompress the neurovascular structures [3]. Thoracic outlet syndrome is often left out of the differential diagnoses in pediatric patients due to the complexity of the symptoms and the notion that children do not develop thoracic outlet syndrome [5]. The symptoms of thoracic outlet syndrome in children differ from those in adults. The most common presentation of thoracic outlet syndrome in pediatric patients is neck discomfort, and numbness, weakness, or sensory loss to the upper extremity [5]. In order to reduce the risk of long-term complications including disability and chronic pain, thoracic outlet syndrome should be quickly diagnosed and treated [3].

Due to the continuous growth of children, compression of the brachial plexus can quickly cause abnormal development of the affected limb. Surgical decompression is considered an absolute treatment for thoracic outlet syndrome and should not be delayed in children with symptomatic thoracic outlet syndrome [5]. However, our patient had a two year delay in treatment. In a study by Rehemutula et.al., 13 pediatric patients were treated for symptomatic thoracic outlet syndrome with either conservative treatments or surgery. Surgical intervention was offered to ten patients that had limb and neck discomfort, decreased muscle strength or atrophy, and decreased sensation to the upper limb. Of these, six patients were able to return to school within two weeks of surgery and there were no reported complications or pain at the patient’s postoperative follow up. Our patient was able to return to competitive gymnastics six weeks after surgery and won a state title that year.

Our patient had several rare diagnoses including an isolated first rib fracture, resultant pseudoarthrosis of the first rib, and the development of symptomatic thoracic outlet syndrome, which occurred after two years of multiple rounds of physical therapy that failed to improve the patient's symptoms. Further appropriate radiologic imaging should have been considered early when the patient repeatedly presented with the same symptoms, instead of the delay that occurred in this case. Careful and thorough serial physical exams also should have been done with each return visit. Removal of the left first rib should have been considered the most logical option with the best probable outcome when it was evident that numerous courses of physical therapy did not resolve the patient’s symptoms.

## Conclusions

Children involved in high impact sports are subject to fractures due to the muscles pulling on the bone. Our patient not only had a first rib fracture, but also had incorrect healing of the fracture leading to pseudoarthrosis and eventual thoracic outlet syndrome. A thorough physical exam with visual examination and palpation performed early likely could have guided further evaluation, leading to earlier treatment. With the continued failure of conservative treatment for pain, more imaging studies should have been ordered sooner to evaluate for any missed pathologies. In retrospect, the left first rib fracture and the first sign of the pseudoarthrosis can be visualized on the initial chest X-ray (Fig. 1). Removal of the first rib was the definitive treatment in this case, as evidenced by the patient having total resolution of symptoms and return to competition shortly post-operatively. This case illustrates the need to consider other pathologies than “overuse syndrome” in a competitive prepubertal athlete with chronic shoulder pain, and especially when early surgical intervention is curative.

## Data Availability

Not applicable.
